# Count Cavour

**Published:** 1862-06

**Authors:** Jno. C. Darby

**Affiliations:** Lexington, Ky.


### HALL'S JOURNAL OF HEALTH.

Our Legitimate Scope is almost boundless: for whatever begets pleasurable and harmless feelings, promotes Health; and whatever induces disagreeable sensations, engenders Disease.

WE AIM TO SHOW HOW DISEASE MAY BE AVOIDED, AND THAT IT IS BEST, WHEN SICKNESS COMES, TO TAKE NO MEDICINE WITHOUT CONSULTING A PHYSICIAN.

Vol. IX.] JUNE, 1862. [No. 6.

### COUNT CAVOUR;

### OR QUACKERY AND HIGH-LIVING.

AN Italian nobleman, one of the very greatest of living statesmen, died recently, at the close of his fifty-second year. America, England, France, and Italy, four nations, sincerely regretted his death, as did the friends of human liberty every where. He ought to have lived at least twenty years longer, to have used the power which long experience, a great intellect, and gigantic political abilities gave him for good, for Sardinia, for united Italy, and for mankind. But he died before his time, while in the very zenith of his power, his efficiency, and his fame, as many great men have done before, in consequence, first, of yielding to the gratification of the animal appetites; and second, by the weak presumption, not uncommon with smaller minds, of prescribing for himself, of being his own physician. The public record of his case is: "He died of congestion of the brain, arising from intense occupation, want of bodily exercise, and either too strong an appetite, or else an excessive indulgence in the pleasures of his well-appointed table." In plainer language, he died of apoplexy, from eating too much and exercising too little. In fact, the great Count could govern a nation better than he could govern himself. His presumption completed the ruin of his manly frame and once vigorous constitution. We have all read of the Roman ruler who loved eating so well, that when he had swallowed as much as his paunch could possibly hold, he would take an emetic, that he might repeat the pleasure. Count Cavour had for a long time been a great feeder; and as he persisted in taking no exercise, he made too much blood, and that, too, of a bad quality; and being a great thinker, this attracted the blood to the brain faster than it could be conveyed away by the sluggish veins; hence, there was such a great accumulation, that the brain was compressed, crowded, and the powers of life were for the moment in a state of suppression. He had found out, in previous milder attacks, that the quickest way to get relieved of the surplus of blood was to open a vein in the arm, and speedily "Richard was himself again." But in this last attack, having no medical knowledge beyond the general fact that bleeding had relieved him before, he inferred that it would do so again, and that the only rule to govern himself by was to let the blood flow until he was relieved, and as often as the symptoms returned. The result was, that he did not send for his physician, but for a professional bleeder, whose whole duty consisted in opening a vein, and letting the blood run until he was ordered to stop it. Hence, neither the bleeder nor the Count's physicians were to blame, because he was in effect bled to death before he consulted a physician at all. It is admitted that "Count Cavour was bled to death." He was bled seven times; but by his own directions, given to a non-medical man.

There are not a few persons who, from having observed that certain good effects followed certain remedies, do not hesitate to administer those remedies under what appear to them to be similar circumstances; and then it is easy to slide into the practice of increasing the quantity or force of the remedy, when the effect is not decided enough. For a few times, or for some considerable time, they are apparently successful, gain self-confidence with great rapidity, and do not hesitate to state, with the utmost complacency, "I believe I know about as much as the doctors do;" and armed with a rag dipped in cold water, or a bottle of red-pepper, or a pint of catnip-tea, or a box of favorite pills, they think themselves invincible practitioners. Count Cavour's great remedy was the lancet; with that at hand, he felt defiance to death and the doctors. Three things he seems not to have known: 1st. That any one remedy, often repeated, begins to lose its power; 2d. That blood-letting, often repeated, tends to increase the frequency of the attacks which it seems to remove; 3d. That two ailments will appear to a general observer precisely identical, yet the remedy which would cure in one case would kill in the other, in consequence of a symptom which only a professional eye would note.

The sum of the whole matter is this, that man is a fool, who, in a grave case of sickness as to himself, his family, or those under his control, fails to call in promptly a regularly educated physician, or one who, if not regularly educated, has had long years of practical and extensive experience; and when death results from such omission, such an one is a constructive murderer.

It is an inexcusable stupidity for any one either to give or take advice in sickness, except from a physician, unless a physician can not be had; it is worse than a stupidity—it is a pitiful impertinence and a crime.

Not long ago a great theologian died in the very prime of his life, before he was fifty. He was said to be one of the most learned divines living. It was also said of him that he "seemed to speak of physicians with a kind of contempt;" and feeling thus, he allowed a trivial disease to progress unchecked, until it became of a fatal character. It killed him on the evening of the very day in which he had ridden out, and when he supposed he was on the point of getting well. It is to be hoped his theology was better than his physic.

The last sentence was scarcely finished, when on opening an exchange, the following was read, in an obituary of a useful citizen, a kind neighbor, and a good man: "He was in the enjoyment of a large portion of health and vigor, and bade fair to live to be very old; but he was attacked with typhoid pneumonia. Being accustomed to prescribe for himself when slightly unwell, and being unaware of the nature of the disease under which he was laboring, he neglected to call a physician, until it was too late to afford him any relief." This was precisely the case with Count Cavour, the William Pitt of Italy, according to the London *Lancet.* He doctored himself until he was really dying, the physician being called in a very few hours before he expired.

We here waive our rule, and give place to an article just received in reference to Cavour's death. It is from the pen of a physician of distinction, who graduated in medicine at the time we did, being of the same class:

"The untimely death of the distinguished Italian statesman, in the prime of life, when his patriotic and valuable services were so much needed by his countrymen, has led me to reflect on the unexpected death of many great and useful men. A large number of men, in the vigor of manhood, and in apparently perfect health, die annually, of whom it may be said, they are well to-day and dead to-morrow. Humanly speaking, these men ought not to die when they do.

"A Turin correspondent, writing a few days before his death, says: 'Count Cavour's indisposition exhibits the symptoms of all his previous attacks, a sudden seizure and a speedy recovery. He was bled four times in three days. The complaint is congestion of the brain, arising from intense occupation, want of bodily exercise, either too strong an appetite, or else excessive indulgence in the pleasures of his well-appointed table. Periodical bleeding had become a necessity with him, as with too many of his countrymen.' Count Cavour was a man of florid complexion, sanguine temperament, full habit, short neck. He loved good-cheer, and even quiet revels, among his intimate friends.

"Physicians bleed with one of two objects: to subdue inflammation, or remove congestion. But there is not a professional bleeder in Italy who knows when to bleed, and how much, to subdue a particular inflammation, or in a case of serious congestion, because this latter pathological condition is properly understood only by eminent medical men. In cases of congestion, depending very much on the action of the heart, and the condition of the brain, a very few minutes may determine whether blood-letting is safe.

"No physician, who knows when he ought to bleed, should ever be absent during the process. In cases of inflammation, Marshall Hall's directions to bleed to fainting, and then stop, are all-sufficient. In cases like that of Count Cavour, a man should not be bled at all; the best remedy is to do nothing. In all such cases which are recoverable, an easy recumbent posture is all that is necessary. Mustard-plasters and friction, with cold applications to the head, may rouse a man sooner than otherwise, and this will satisfy friends, which is important.

In almost all such cases, when a vein is opened, the blood will flow freely for a few seconds, and then ceases. In a man with Cavour's habits of life, the supposed congestion of the brain is simulated, not real. Great mental exertion does not produce that pathological condition known to medical men as congestion of the brain; it is rather torpor of the brain, the proximate cause of which is reflex nervous disturbance, induced by the debility of an over-loaded, over-taxed stomach. As soon as the symptoms of the apparent congestion pass off, so soon as the torpor of the brain begins to disappear, if there is reason to believe that the stomach contains undigested food, (and if the attack is soon after a full meal, you may be sure such is the case,) give a glass of warm water in which has been stirred two teaspoonfuls each of common table-salt and ground mustard, to vomit freely; then administer some remedy which will act certainly, speedily, and powerfully on the liver; because high living, with the liberal use of good wines or alcoholic liquors, such as do not disturb the stomach primarily, as all bad liquors do, narcotize the blood, unless the individual takes a good deal of hard out-door exercise. English gentlemen live high, and enjoy good health, [apparently, for a time.—Ed.,] although they are scholars and statesmen, because they exercise much on horseback. For a longer or shorter time, depending very much on the climate, good wines and pure alcoholic liquors stimulate the liver, and when taken in moderation, improve the health; but sooner or later they render the liver torpid, healthy bile is not secreted, the effete matter of the body remains in the blood, rendering it less vital, while at the same time the direct narcotic effect of the wine and brandy is exerted on the brain and nervous system, weakening them. Abstinence from these drinks, with abundant out-door exercise, is the best remedy and the best preventive of all such cases. But where the man will not take the exercise, (many scholars, statesmen, and professional men think they have not time to take it,) and still will live high, then some medicine which will act on the liver is the only safe reliance. There are vegetable remedies which will keep the liver acting, and also various mineral waters, when drunk from the springs, fresh and pure; but calomel is not only the most efficient and reliable, but it is the safest, mildest remedy which can be used. But to do any good, either as a preventive or for immediate relief, it must be given in efficient doses, from twenty to sixty grains at a time, according to the threatening symptoms of the case, because in these cases the seats of the trouble are in the liver and the stomach—the latter for the most part temporarily, as in a fit, the former permanently. The stomach must be relieved at once by an emetic, as was the practice among the old Romans, and then have rest; next the liver must be made to perform its physiological functions of keeping the blood pure, by removing the effete matter which is emptied into the blood by the expletory absorbents every minute of a man's life. The malady in such cases is functional, and the vital functions are all weakened, because the assimilating and excreting organs are prevented from doing their constant work. The disturbance and confusion about the brain is not a congestion, pathologically speaking—it is a torpor. Bleeding may, and sometimes does, give temporary relief; but it is a dangerous remedy, because it is deceptive, for only a small quantity of blood will flow usually, while that which remains is as impure as ever. The system not being in a condition to bear blood-letting, (as in a case of true congestion, where the pulse becomes fuller and stronger as the blood flows freely,) syncope is soon induced, or a decided tendency to it, producing a real congestion, (but one not to be bled for,) and the patient is left exhausted; the true cause of danger is overlooked, the medicines to relieve it are not thought of, and bleeding is again repeated, to remove a condition which every repetition contributes to reproduce. Speaking as a medical man, I would say, if Count Cavour had not been bled at all in his last illness, but had had his stomach emptied, and his liver set to work with promptitude and energy, he would have been well to-day. Of all remedies used in such cases, (and they are numerous, very many of them being among the most efficient men in the community,) blood-letting and opium are the most destructive; but the most fatal field of the latter is in that class of cases where too much strong drink is the cause of the disturbance of the brain. JNO. C. DARBY.

"LEXINGTON, Ky., Jan. 2, 1861."

This is a most interesting subject, and is of incalculable practical importance, because it has a special application to our greatest men. Cavour, Douglas, and Webster were all great men, a head and shoulders above the millions of their own times, and the manner of their lives, their last illness, and their death was remarkably similar. All had been accustomed to drink freely, to "live high," according to accepted reports; all were for the most part insensible for some days, with only an occasional and instantaneous gleam of intelligence, and their great minds went out in the night of insensibility. In either case, there were millions of men who would have given all they possessed in the world to have known the true nature of their maladies, and to have possessed the means of their cure. And as there are multitudes of other great men who are destined to the same habits of life, the same kind of illness, and the same death of mental darkness, the unprofessional reader may be warned hereby as to opium, alcohol, and tobacco. Touch not, taste not, handle not, as you value your health, your life, your mind. At the same time, the educated physicians who regularly read this JOURNAL may make a use of the suggestions of this article, which will be of great practical value as long as they live.

It is too often the fate of distinguished persons, that when they are ill, the judgment of the attending physician becomes so disturbed by the sense of his responsibility, that the chances of success are not half as great as if the patient had been a day-laborer. It is said that Sir Robert Peel was allowed to die because it "hurt him" to adjust the broken bone. Mr. Webster was killed by a fall from his carriage. He did not, it is true, immediately die, but he was never well afterward; his nervous system received a shock from which it never recovered, and when he became confined to his bed, the impression was made that he was suffering from some ailment of the stomach. "Cancer" of that organ was stated to be the malady, and as persons never recover from it, the public mind reconciled itself to an inevitable necessity. But a *post-mortem* examination exhibited the fact that there was nothing like cancer in the stomach; but there was a substance in the liver "as black as tar," which was nothing more than vitiated bile, which with very great certainty could have been carried off from the system by a timely exhibition of efficient hepatic remedies. With equal certainty Mr. Douglas could have been saved in the same way. The fact is, Northern physicians have such an apparent horror of medicine, or of a public opinion vitiated by the senseless jargon of water-cure journals, steam-doctors, vegetarians, spiritualists, *et id omne*, that they are daily letting their patients die by default, or slide helplessly into the current of any popular delusion which happens to prevail at the time, such as nitrate of silver, cod-liver oil, the phosphates, brandy, and Bourbon whisky. Nearly every patient coming to us lately from New-England has been swilling liquor by advice of the "family physician." Within a month a lady came to us from down-East, who was taking, by advice of a regular physician, black-drop, cherry pectoral, the phosphates, cod-liver oil, and half a pint of Bourbon whisky a day! It does seem to us that New-England, with all her boasted intelligence, is getting no better fast, both in her theology and her physic. Is the Boston *Medical and Surgical Journal*, the medical organ of New-England, asleep? Is the Hub of the Universe "chocked"? Or are these results the legitimate unfolding of their teachings? Is the acme of medical knowledge reached? Is the biggest gun—the last resort when all other things fail—a plug of opium, a bottle of "Cherry Pectoral," or a jug of whisky? We pause for a reply!

